# Does school-based physical activity decrease overweight and obesity in children aged 6–9 years? A two-year non-randomized longitudinal intervention study in the Czech Republic

**DOI:** 10.1186/1471-2458-12-570

**Published:** 2012-07-29

**Authors:** Erik Sigmund, Walid El Ansari, Dagmar Sigmundová

**Affiliations:** 1 Center for Kinanthropology Research, Institute of Active Lifestyle, Faculty of Physical Culture, Palacky University in Olomouc, Olomouc, Czech Republic; 2 Faculty of Applied Sciences, University of Gloucestershire, Gloucester, United Kingdom

## Abstract

**Background:**

Globally, efforts aimed at the prevention of childhood obesity have led to the implementation of a range of school-based interventions. This study assessed whether augmenting physical activity (PA) within the school setting resulted in increased daily PA and decreased overweight/obesity levels in 6-9-year-old children.

**Methods:**

Across the first to third primary school years, PA of 84 girls and 92 boys was objectively monitored five times (each for seven successive days) using Yamax pedometer (step counts) and Caltrac accelerometer (activity energy expenditure AEE - kcal/kg per day). Four schools were selected to participate in the research (2 intervention, 2 controls), comprising intervention (43 girls, 45 boys) and control children (41 girls, 47 boys). The study was non-randomized and the intervention schools were selected on the basis of existing PA-conducive environment. Analyses of variance (ANOVA) for repeated measures examined the PA programme and gender effects on the step counts and AEE. Logistic regression (Enter method) determined the obesity and overweight occurrence prospect over the course of implementation of the PA intervention.

**Results:**

There was a significant increase of school-based PA during schooldays in intervention children (from ≈ 1718 to ≈ 3247 steps per day; and from 2.1 to ≈ 3.6 Kcal/Kg per day) in comparison with the control children. Increased school-based PA of intervention children during schooldays contributed to them achieving >10,500 steps and >10.5 Kcal/Kg per school day across the 2 years of the study, and resulted in a stop of the decline in PA levels that is known to be associated with the increasing age of children. Increased school-based PA had also positive impact on leisure time PA of schooldays and on PA at weekends of intervention children. One year after the start of the PA intervention, the odds of being overweight or obese in the intervention children was almost three times lower than that of control children (p < 0.005), and these odds steadily decreased with the duration of the intervention.

**Conclusions:**

The findings suggest that school-based PA (Physical Education lessons, PA during short breaks and longer recesses, PA at after-school nursery) in compatible active environments (child-friendly gym and school playground, corridors with movement and playing around corners and for games) has a vital role in obesity and overweight reduction among younger pupils.

## Background

The increase in overweight and obese children is a global concern [1-5]. Indeed, children’s low physical activity (PA) levels [5,6], together with the increase in their sedentary behaviours [5] have collectively prompted research into strategies and programmes that could enhance the PA levels [7,8] in order to alleviate the increase in obesity amongst children [3,9-11].

School environments and settings offer many opportunities for PA intervention programmes aimed at young pupils [12,13]. Children spend considerable proportions of their day within the school [14], with potential occasions that could enable the development of healthy lifestyle habits [13,15]. Hence, PA associated with school e.g. physical education [15,16] and PA during recess periods, lunch breaks or after school nursery [16-19] are viewed as major options for school aged children to increase or achieve their recommended daily PA [15,20].

During childhood and adolescence, regular PA helps to maintain a healthy body weight; is associated with the positive development of healthy musculoskeletal and cardiovascular systems, as well as neuromuscular awareness; and is being promoted as an objective for disease prevention [3,8,14,15]. Despite that school-aged children’s PA is mostly undertaken outside of the school environment [16,18,20], school-based PA is an irreplaceable contributor to the overall PA on schooldays that plays a part in the achievement of PA guidelines for maintaining health [20,21]. Whilst the positive association between school-based PA and leisure time PA has been confirmed [7] even in overweight-to-obese school-aged children [14], however, this positive association between school-based PA and weekends PA is still not well investigated. PA of school-aged children and teenagers is lower at weekends than during schooldays [19,22], but detailed analyses of school-based, leisure time and weekend PA within PA intervention programmes still requires further research.

Globally, efforts aimed at the prevention of childhood obesity have led to the implementation of a range of school-based interventions [10,11,23-26]. Nevertheless, the heterogeneity of studies renders it difficult to draw generalizations about the intervention/s that were most effective [24,25,27]. Yet, despite the methodological diversities as well as the geographic, climatic, ethnic, conceptual and regional (country) characteristics associated with various PA interventions implemented in different countries, it is fortunately possible to outline some features of effective interventions that aim to decrease obesity levels of primary school children. For instance, combination/s of increased PA (decreased sedentary behaviours) and appropriate diet appears to be more effective in reducing obesity than either increased PA alone or an appropriate diet alone [10,24,26]. Similarly, long-term (>1 year) interventions stand stronger chances of reducing obesity than shorter-term (<1 year) interventions [11,13,24]. Furthermore, gender-specific interventions appear to be more effective in reducing obesity than general interventions [23]. In addition, parents’ participation in intervention programmes increases the chance of successful obesity reduction [23,28,29]; and, a compatible active environment together with the availability of various game equipment triggers PA in children [30-32].

Given that there is no universal intervention that has demonstrated a long-term PA increase in children whilst simultaneously reducing their obesity levels, there have been calls for research on strategies that could increase PA and alleviate obesity in children [9-11,30]. However, whilst longitudinal studies of school-aged children to reduce overweight/ obesity by increasing the school-based PA have been implemented in Western countries [33-35], there is a notable lack of such longitudinal studies in Central and Eastern European nations (e.g. in the Czech Republic). Similarly, across these previously Eastern-Block countries, there is lack of research of interventions aimed at the long-term increase of PA in children that simultaneously addressed the issues of gender-specific interventions, parents’ participation in intervention programmes and the availability of compatible active environment and game equipment. Indeed, longitudinal studies (3 years’ duration) comprising repeated monitoring (twice a year, total of five times) that includes schooldays and weekends (seven successive days monitoring each time) using objective measures (accelerometer and pedometer) of PA in 6-9-year old children are rare in Eastern Europe. This is despite that policy makers require evidence about the effectiveness of PA interventions in order to guide planning. The study described in this paper bridges this gap, and assesses the influence of school-based PA (that is mostly gender specific, with parent’s participation, and in a conducive environment with available equipment) on overweight and obesity levels in children in the Czech Republic.

### Aim of the study

This study assessed the effectiveness of a school-based two-year PA intervention in reducing obesity and overweight in 6–9 year-old children over the course of the first to the third primary school years (from 1^st^ Grade to 3^rd^ Grade primary school). The specific objectives were to:

Describe and compare the PA levels of the intervention and control groups of girls and boys before, during, and at the end of intervention;

Compare the levels of schooldays and weekends PA of the intervention and control girls and boys;

During schooldays, compare the levels of school-based and leisure time PA of the intervention and control girls and boys;

Describe and compare the proportion of overweight and obese children in the intervention and control girls and boys before, during, and at the end of the intervention; and,

Express the effect of participation in the PA intervention on overweight and obesity levels of the children.

## Methods

### Participants and settings

The Institutional Research Ethics Committee at the Faculty of Physical Culture, Palacky University approved the study. All potential participants were provided with information outlining the study aims and objectives, and children’s and parents’ participation was voluntary (no financial incentives were provided). The current study expands upon earlier longitudinal research in the Czech Republic of changes in PA of 176 (84 girls; 92 boys) pre-schoolers (kindergarten) and first-grade (first year of primary school) children at four primary schools (2 intervention and 2 control schools) in two regional cities (Olomouc and Prostejov) in the Moravia region, Czech Republic [19]. This earlier longitudinal research [19] highlighted a significant decrease of school time PA after the transition of children from kindergarten to 1^st^ grade of primary school. The current longitudinal study deals with changes of PA and body weight of children during their transition from 1^st^ to 3^rd^ grade of primary school, and hence builds upon and extends the temporal span where the previous research [19] ended. Written informed consent was obtained from parents of all children participating in the study.

The two intervention schools were selected based on their participation in the regional “Healthy Schools” project which brings together schools that: focus on health behaviours; and, support school based PA of their children (including after school nursery primarily focussed on PA and games) [36]. The “Healthy Schools” project was developed by World Health Organization for Europe (the Ministry of Education, Youth and Sports in the Czech Republic adopted the project in 1991) in response to the increased unhealthy behaviours in school aged children. The “Healthy Schools” project included many activities/ programmes (e.g. healthy diet habits, drug prevention, sports and singing competitions, poetry reading contests, school trips, and PA programmes). The PA intervention presented in this paper is a component of the PA programmes of the “Healthy Schools” project. The two selected intervention schools had to meet the same four criteria (sports and singing competitions, poetry reading contests, school trips, and PA programmes) of the “Healthy Schools” project. Both intervention schools had similar PA-conducive environments: a gymnasium, grass playground and yard, sports field, basketball court, corridors and corners conducive of movement and playing, and rooms for table and board games (tennis, football, hockey). In contrast, the two control schools were not participating in the “Healthy Schools” regional project, had less PA-conducive environments (only one small gymnasium and playground, and standard corridors without special PA corners and rooms), and the orientation of their after-school nursery was not primarily concentrated on PA and games.

In both the intervention and control schools, shortly after the children started attending their first year at primary school (September 2006), their baseline measurements were undertaken (their baseline weekly PA was monitored). Then (October 2006), the control schools continued with their traditional ‘standard’ PA programmes; whilst a PA intervention was launched at the two intervention schools (implemented in addition to the traditional ‘standard’ PA programmes) (described below). At the intervention schools, the school teachers and the research team organised the PA intervention programme, in collaboration with students of the Physical Culture and Pedagogical Faculties at Palacky University. Children’s participation in the intervention was supported by their parents who co-operated with the research team in recording their child’s PA/ sedentary behaviour data in the child’s PA log book (described below); and also assisted the research team in explaining to the children the role of PA and active lifestyles in the prevention of obesity.

### Standard PA programme and PA intervention programme

The standard PA programme (implemented in control and intervention schools) comprised mandatory two 45-minute physical education (PE) lessons per week (boys and girls together) undertaken in the gym/ playground. The PE focussed on overall physical development through movement games (tag, games based on locomotion in rows/ circles, simplified versions of dodge-ball/ football), simple gymnastic exercises (squats, sit-ups, bounces, etc.), and exercises with equipment e.g. ball (dribbling, throwing at a target, catching), skipping rope (jumping over), hoop (running, rotating, going through), or benches (walking and different kinds of jumping over). Further, at the control schools, children could also undertake additional PA in recess periods and at an after-school nursery if they wished to, subject to availability of school equipment and teacher’s choice, or alternatively could choose some other sedentary activity (e.g. drawing or doing homework).

In addition to the standard programme described above, the PA intervention (intervention schools only) comprised: 1) one 20-minute recess with PA content (in gym/ school playground); 2) PA (playing) undertaken during after-school nursery (≈40 minutes to ≤ 90 minutes); and 3) an average of 2–3 short breaks per day (lasting 3–5 minutes each, in between lessons) were PA could be carried out in the corridors with movement and playing around corners and/or rooms for table and board games that were close to the classes. Table 1 depicts the schooldays’ PA content at the intervention and control schools.

**Table 1:** Schooldays PA content of intervention and control schools from October 2006 to September 2008

		**Intervention Schools**	**Control Schools**
	Gender-specific	Girls and boys separately choose type, equipment and content of activities during co-educational teaching	Girls and boys girls undertake together same type and content of activities during co-educational teaching
**Type (duration)**	**Frequency**	**Description and Examples**	
**PE lessons**	2 per week	Overall physical development though movement games, simple gymnastic exercises, and exercises with equipment in coeducational teaching	
(45 minutes)			
		**Primary focus on increased PA content**	**General content Orientation**
**Short breaks**	2-3 per day	Movement playing in classroom/ room for table and board games	Painting, drawing, writing in classroom
(3–5 minutes)			
**Recess**	3-4 per week	Movement playing in corridors/ room for table and board games	Painting, drawing, writing in classroom
(20 minutes)			
**After-school nursery**	each day	Movement games, playing, gymnastic exercises, exercises with equipment in gym/ school playground	Painting, drawing, singing, doing homework, reading, playing board games in classroom
(≈40-90 minutes)			

PE: physical education; PA: physical activity.

At the intervention schools, both the recess and after-school nursery active playing comprised individual and group games and exercises with equipment (skipping ropes, hoops, foam, soft and volleyball balls, overballs, soft-tennis and badminton rackets, baseball bats, hopscotch, balls and rubbers, scooters, children scooters, Frisbee, basketball hoops, ropes, wall bars), age-adjusted games (football, floorball, volleyball, dodge-ball, table tennis), and movement games (tag, games with a circular cloth, nursery rhymes with movement). The girls and boys were free to change the type and intensity of the PA, as the PA content was based upon participants’ preferences/ capabilities, climate conditions and available teachers (in accordance with their curricula). A feature of this PA intervention was that it was gender-specific – one of the teachers organised the PA programme for girls; and another teacher organised it for boys. Hence, children were free to play girls and boys together in couples, threesomes and small groups. However, if the children wished, same-gender playing was not prohibited by research team. All types of PA performed in the PE lessons, short breaks, and recesses, and at the after-school nursery were organized under the umbrella of collective, co-education teaching. Co-education teaching denotes the teaching of both girls and boys in the same school, in the same classes and through the same courses of study programme. In summary, the focus was on children’s active participation.

### PA monitoring, and determining overweight and obesity

Over 2006–2008, participants’ free-living PA was measured on regular basis (five times, seven successive days each time) during September and April (Table 2).

**Table 2:** **School term dates of PA monitoring, numbers and age of participating children by gender - 1**^**st**^**Grade through 3**^**rd**^**Grade**

		**1**^**st**^**Grade**		**2**^**nd**^**Grade**		**3**^**rd**^**Grade**
		**September 2006**	**April 2007**	**September 2007**	**April 2008**	**September 2008**
**Term Dates (day.month)**		5.9 - 26.9	11.4 - 27.4	10.9 - 26.9	8.4 - 29.4	4.9 - 25.9
**Number (age)**						
Intervention	Girls	43 (6.9±0.4)	43 (7.5±0.4)	43 (7.9±0.4)	43 (8.5±0.4)	43 (8.9±0.4)
	Boys	45 (6.6±0.6)	45 (7.2±0.6)	45 (7.6±0.6)	45 (8.2±0.6)	45 (8.6±0.6)
Control	Girls	41 (6.8±0.5)	41 (7.4±0.5)	41 (7.8±0.5)	41 (8.4±0.5)	41 (8.8±0.5)
	Boys	47 (6.6±0.5)	47 (7.2±0.5)	47 (7.6±0.5)	47 (8.2±0.5)	47 (8.6±0.5)

Measurement was undertaken using a standardised method of continuous monitoring of daily PA that comprised: Caltrac accelerometer (Muscle Dynamic Fitness Network, Torrance, CA, USA); Yamax Digiwalker SW-200 pedometer (Yamax Corporation, Tokyo, Japan); and, a PA log book for inputting the Caltrac and Yamax data [19].

The Caltrac accelerometer is a light, pocket instrument that scans vertical movement [37]. A built-in ceramic crystal transfers kinetic acceleration into electrical impulses which can be subsequently recalculated (accounting for somatic features e.g. body mass, height, age, sex) into energy output units [kcal] [38]. We quantified the PA levels through the variable activity energy expenditure (AEE) which represents the net value of energy of a given PA, i.e. total energy expenditure minus the resting metabolism [39]. In determining AEE value, the Caltrac uses the following equation to calculate resting metabolism based on the subject’s age, height, weight and gender [40,41]: female [kcal/min] = ((331·weight [lb]) + (351·height [in.]) – (352·age [years]) + 49 854)/100 000; and male [kcal/min] = ((473·weight [lb]) + (982·height [in.]) – (531·age [years]) + 4686)/100 000. For group comparisons of girls and boys with different body weights, it is appropriate to use relative AEE values, calculated to one Kilogram of the participant’s weight (Kcal/Kg·day^-1^ or Kcal/Kg·hour^-1^) [39]. In order to ascertain the daily energy expenditure in children, the Caltrac accelerometer was validated to a single-day heart-pace recording (r_P_ = 0.40–0.54, p < 0.02) with high (r_P_ = 0.96) internal-group reliability [37,41]. Due to the significant agreement (e.g. in walking) between energy expenditure from Caltrac and indirect calorimetry (r_P_ = 0.80 p < 0.001), and between Caltrac and VO_2_ oxygen consumption (r_P_ = 0.85 p < 0.001), this type of accelerometer is recommended for daily energy expenditure detection in children [42,43]. Hence for outcome consistency and also parents’ abilities to handle the apparatus, we used the Caltrac accelerometer for continuous monitoring of PA.

The Yamax Digiwalker SW-200 is a commercially available, small and light electronic pedometer measuring vertical oscillations. Its circuit switches on and off through a pendulum arm that moves with the vertical oscillations of walking [44]. Every vertical oscillation stronger than the apparatus’s threshold (0.35 g) is considered a step [45]. The total amount of steps and consequently the calculated distance, and AEE, are depicted on the display. Pedometers are most accurate in counting the number of steps, less accurate in calculating distance, and least precise at estimating energy expenditure [46]. Hence, in line with others [47], we employed the step counts as the pedometer outcome variable.

The somatic features of the participants were measured 2–7 days prior to the start of monitoring in order to adjust the individual settings of the Caltrac accelerometer (we inputted participant’s gender, age, body weight, and body height), and also for preparation of the individual PA log books (we inputted participant’s name, days and dates of monitoring). Participant’s calendar age was calculated from date of birth until first monitoring day. The research team measured the body height and body weight of participants (Anthropometer A-319 - Trystom, Olomouc, Czech Republic; Tanita WB 110 S MA - Quick Medical Corporation, Seattle, WA, USA respectively) to nearest 0.5 cm and 0.1 kg on the morning of the first lesson of the first day at primary school. BMI was calculated as body mass [kg] divided by height [m] squared. Obesity, overweight and normal body mass were classified using percentile BMI graph for girls and boys aged > 5–19 years [48], where overweight and obesity represented the 85–97 and > 97 percentiles respectively of age-differentiated BMI.

The monitoring of PA was in line with previous research of kindergarten and first grade school children [19]. On our first monitoring day, each participating child received an elastic belt with two pockets (for accelerometer and pedometer), along with an individual PA log book. The belt ensured tight placement of the devices on the right hip during the daily PA monitoring. Children were instructed to wear the belt with both devices for at least eight hours per day (with exception of rest, sleep and bathing). The research team trained the participating teachers and parents to appropriately: 1) operate the accelerometer and pedometer; 2) read the values expressed by each device; and, 3) record the values into the child’s individual PA log books. Participant’s teacher/s and parents recorded the data in the PA log book which comprised three sections: the AEE (from accelerometer); the achieved step counts (from pedometer); and the third section was the composition of the PA that was undertaken, its duration, intensity and type. The measured AEE values [kcal] and step counts were recorded in the PA log book four times each day (after getting up - by parent; after arriving at and before leaving school - by teacher; before sleep - by parent). Monitors were not reset throughout the day. On the morning of the first monitoring day, after each participating child received an elastic belt and an individual PA log book, we reset the values on the monitors’ displays and entered the first record (zero values) of AEE and step counts into the individual’s PA log book. After that, participant’s teacher/s and parents recorded the data in the PA log book continuously throughout the weekly PA monitoring.

### Statistical processing and data interpretation

Data were analysed using STATISTICA v.9 and SPSS v19. Four two-way (intervention and control group × 2 genders) analyses of variance (ANOVA) for repeated measures examined the PA programme and gender effects on PA levels, separately for the amount of steps and AEE. Schooldays, weekends, school and leisure times of working days were used as dependent variables to thoroughly examine the PA programme and gender effects on PA levels in each part of the monitored week. Tukey’s HSD post-hoc test identified differences in PA levels between control and intervention children at different times of week (schooldays × weekends), and time of day (school × leisure time). Data were adjusted only for clustering at school level due to the same design of PA intervention programme and also due to the similar PA-conducive environments at the selected intervention schools. When using ANOVA for repeated measures, clustering was controlled for employing the school attendance list and PA log book. *T*-test for dependent samples identified differences of the PA levels in each of the repetitive measures in participants of the same sex and group (i.e. either control or intervention). Logistic regression (Enter method) determined the obesity and overweight occurrence prospect over the course of implementation of the PA intervention. The model included independent variables such as affiliation with a group (intervention vs. control) and sex (girls vs. boys). The strength of the relationships between the independent (affiliation with a group, sex) and dependent (AEE and amount of steps) variables on schooldays, weekends, school time and leisure time was assessed by means of “effect size” d coefficient for repetitive measures [49], where values d = 0.2, 0.5 and 0.8 may be interpreted as minor, middle and major effects [50,51].

## Results

### Baseline – Before the start of PA intervention (beginning of September 2006)

**Figure 1:**
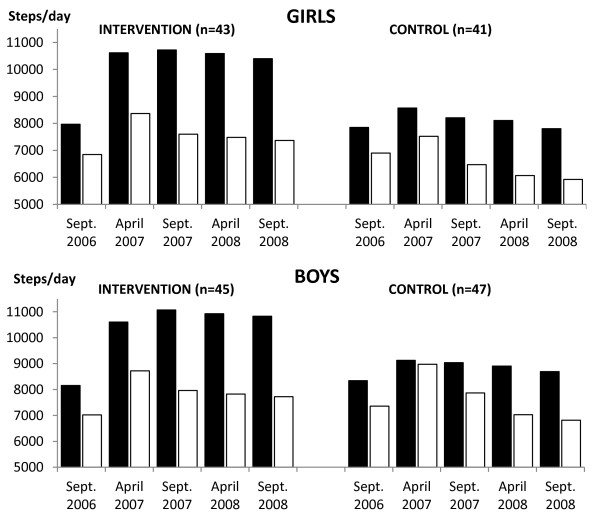
**Mean daily steps counts of intervention and control children across the two-year PA programme.** PA - physical activity; ▪ Schooldays; □ Weekends.

**Figure 2:**
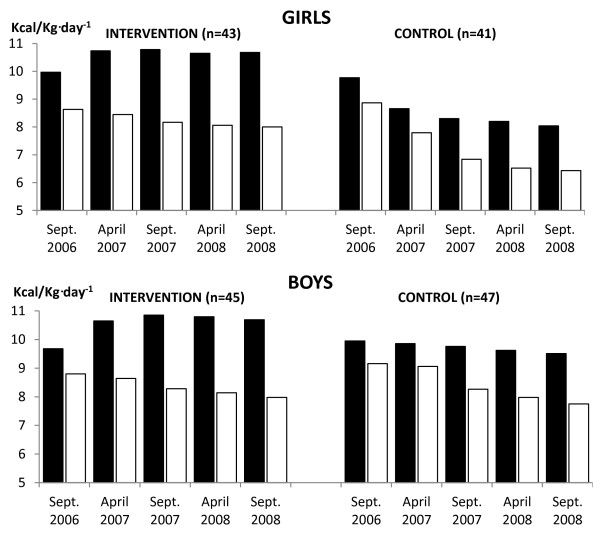
**Mean daily AEE (Kcal/Kg·day**^**-1**^**) of intervention and control children across the two-year PA programme.** AEE - activity energy expenditure; PA - physical activity; ▪ Schooldays; □ Weekends.

Before the start of intervention, for both genders, there were no differences between the intervention and controls, on schooldays and on weekends, as regards the mean daily step counts (Figure 1) and AEE (Figure 2). Schooldays PA comprised the sum of school time PA and leisure time PA (i.e. time after the after-school nursery).

**Figure 3:**
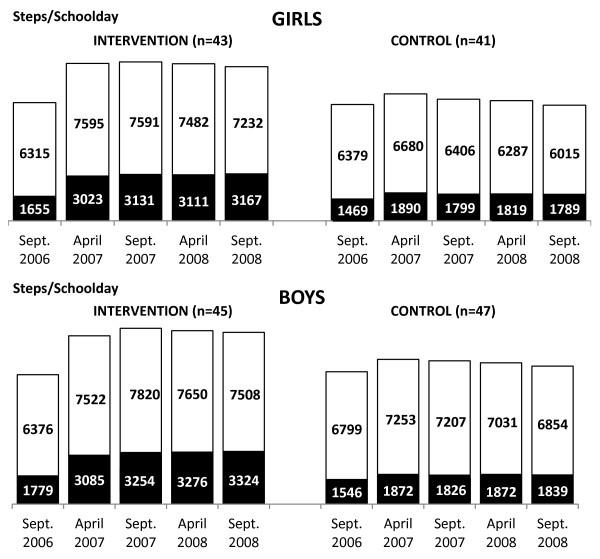
**Mean schooldays steps counts of intervention and control children across the two-year PA programme.** PA - physical activity; ▪ School time; □ Leisure time.

**Figure 4:**
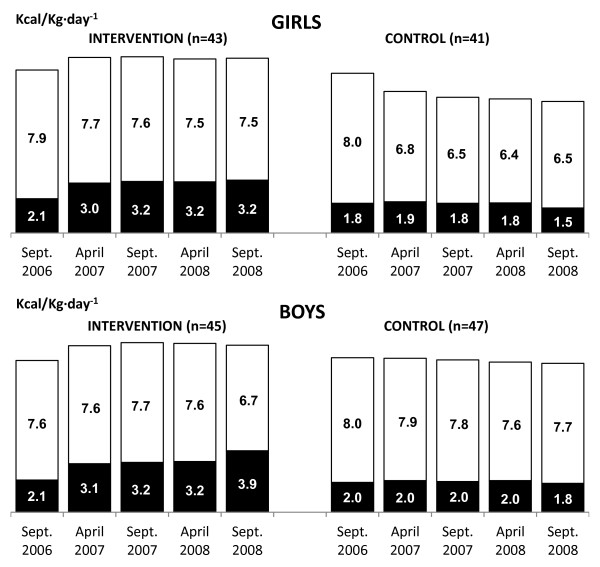
**Mean schooldays AEE (Kcal/Kg·day**^**-1**^**) of intervention and control children across the two-year PA programme.** AEE - activity energy expenditure; PA - physical activity; ▪ School time; □ Leisure time.

**Figure 5:**
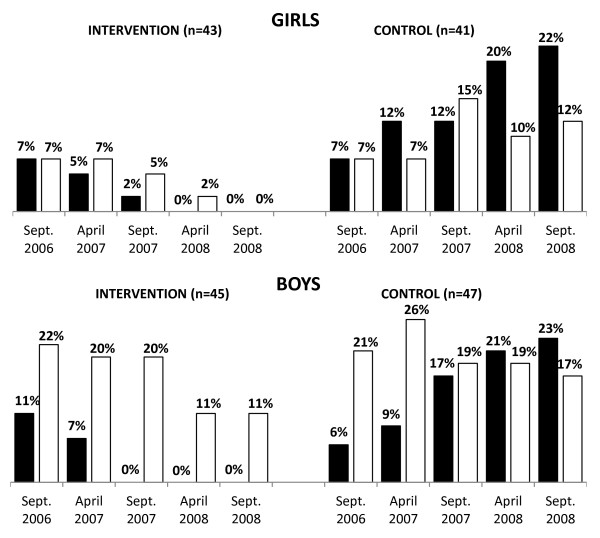
**Mean percentages of obese and overweight children in intervention and control children across the two-year PA programme.** PA - physical activity; ▪ Obese; □ Overweight.

Similarly, before the intervention, for both genders, there were no differences between the intervention and controls, in the school time number of steps (Figure 3) or AEE (Figure 4), and in the leisure time number of steps (Figure 3) or AEE (Figure 4). Furthermore, before the start of the PA intervention, there were no differences in the proportions of obese girls and boys in the intervention (7 % girls; 11 % boys) and control groups (7 % girls; 6 % boys) (Figure 5).

### During the PA intervention (October 2006 - September 2008)

After baseline monitoring of weekly PA and classification of participants’ BMI in accordance with the percentile BMI graph, the PA intervention was launched in the intervention schools, while controls continued with the standard PA programme.

#### *Schooldays PA*

A repeatedly significant positive intervention effect of the PA programme was found for steps per day and AEE (Kcal/Kg·day^-1^) for intervention children (F_STEPS_ = 651.69, p < 0.0001, d = 1.07; F_AEE_ = 91.29, p < 0.0001, d = 0.82) than the control children. The level of schooldays PA of intervention children was repeatedly higher during October 2006 to September 2008 in comparison with the controls’ PA level (Figures 1,2). Gender had a repeatedly significant effect on the level of schooldays PA (F_STEPS_ = 258.19, p < 0.0001, d = 0.21; F_AEE_ = 23.87, p < 0.0001, d = 0.31). However, the effect of gender was more than twice (for AEE) and more than three times (for step counts) lower than the effect of the PA programme. In addition, as regards to the main repeatedly significant effect, there were significant interactions between the PA programme and gender (F_STEPS_ = 5.83, p = 0.0006, d_♀_ = 1.43, d_♂_ = 0.86; F_AEE_ = 4.68, p = 0.0031, d_♀_ = 1.31, d_♂_ = 0.47). On schooldays, intervention girls were more physically active than both the control girls and also the control boys (Figures 1,2). At the end of the PA intervention programme, there was a slight decrease of the proportion of intervention children (girls: 32.5%_APRIL2007_, 33.7%_SEPT2007_, 31.4%_APRIL2008_, 23.5%_SEPT2008_ and boys: 16.7%_APRIL2007_, 18.8%_SEPT2007_, 17.7%_APRIL2008_, 14.4%_SEPT2008_) who met national Czech PA guidelines for maintaining health for children aged 6–11 years (steps per day – 12,000 girls and 14,000 boys; AEE – 11 Kcal/Kg·day^-1^ for girls and 13 Kcal/Kg·day^-1^ for boys) [35]. As for controls, there was a progressive decrease of the proportion of children who achieved these national PA guidelines (girls: 11.0%_APRIL2007_, 9.8%_SEPT2007_, 7.3%_APRIL2008_, 6.1%_SEPT2008_ and boys: 11.7%_APRIL2007_, 8.5%_SEPT2007_, 7.4%_APRIL2008_, 6.4%_SEPT2008_).

#### *School time and leisure time PA*

Only the PA programme had repeatedly significant effect on school time PA level (AEE and steps) during the current school-based PA intervention. Intervention children had significantly higher step counts and AEE at school time than controls (F_STEPS_ = 371.08, p < 0.0001, d = 1.28; F_AEE_ = 4.67, p < 0.0001, d = 1.03) (Figures 3–4). No other significant interaction effects during school time were observed. During the leisure time of schooldays, a significant positive effect of PA programme and gender on step counts was identified (F_PAprogramme_ = 185.57, p < 0.0001; F_GENDER_ = 131.70, p < 0.0001). In addition to the main repeatedly significant effect, there were significant interactions between the PA programme and gender (F_STEPS_ = 2.65, p = 0.05, d_♀_ = 0.73, d_♂_ = 0.28). At leisure time, intervention girls had step counts that were higher than those of both control girls and also control boys (Figures 3–4).

#### *Weekends PA*

Both intervention and control children repeatedly achieved lower daily step counts and AEE during weekends than during schooldays (Figures 1–2). Nevertheless, on weekends, a repeatedly significant positive intervention effect of PA programme was observed for daily step counts and AEE (Kcal/Kg·day^-1^) for intervention children (F_STEPS_ = 629.43, p < 0.0001, d = 0.27; F_AEE_ = 169.61, p < 0.0001, d = 0.20) than controls. No other significant interaction effects at weekends were identified.

Over the course of the PA intervention, the proportions of obese or overweight participants declined in the intervention girls and boys, as opposed to the controls, where the opposite tendency was observed (Figure 5). Nevertheless, a significant decline in obesity and overweight in the intervention children was achieved no sooner than during the second grade of primary school (Sept. 2007) (Table 3).

**Table 3:** Impact of participation in PA intervention on odds of child obesity/overweight combined

	**n**	**1**^**st**^**Grade**				**2**^**nd**^**Grade**				**3**^**rd**^**Grade**	
		**September 2006**		**April 2007**		**September 2007**		**April 2008**		**September 2008**	
		**OR**	**CI**	**OR**	**CI**	**OR**	**CI**	**OR**	**CI**	**OR**	**CI**
**Group**											
Control	88	Ref		Ref		Ref		Ref		Ref	
Intervention	88	1.17	0.57-2.40	0.64	0.31-1.32	0.34^*^	0.16-0.72	0.13^‡^	0.05-0.34	0.09^‡^	0.04-0.27
**Gender**											
Girls	84	Ref		Ref		Ref		Ref		Ref	
Boys	92	2.64^*^	1.24-5.62	2.38^*^	1.13-5.01	1.99	0.94-4.20	2.02	0.91-4.49	1.85	0.83-4.12
**R**^**2**^		0.05		0.06		0.10		0.21		0.25	

N: number; OR: odds ratio; CI: confidence interval; statistical significance *p < 0.005, ^‡^p < 0.001; R^2^: Nagelkerke coefficient of determination, logistic regression model, Enter method.

### End of the PA intervention programme (end of September 2008)

At the final PA monitoring (end of the PA intervention), there was a slight decline in both the schooldays daily step counts and AEE in intervention children (t_♀STEPS_ = 30.03, p < 0.0001, d = 0.11; t_♀AEE_ = 0.79, p = 0.4356, d = 0.02; t_♂STEPS_ = 13.61, p < 0.0001, d = 0.04; t_♂AEE_ = 2.07, p = 0.0442, d = 0.05) and controls (t_♀STEPS_ = 12.48, p < 0.0001, d = 0.18; t_♀AEE_ = 7.95, p < 0.0001, d = 0.09; t_♂STEPS_ = 6.81, p < 0.0001, d = 0.10; t_♂AEE_ = 2.36, p = 0.0226, d =  0.05), in comparison with the results of the precedent measurement (April 2008) (Figures 1–2). In particular, the intervention group’s decline in PA was during the school days’ leisure time, while the controls demonstrated the decline in PA during school time (Figures 3–4).

Based on the percentile BMI graph, after the two-year PA intervention (September 2008), the intervention group did not exhibit any obesity, while about one fifth to one fourth of controls were obese (22% girls and 23% boys). Moreover, after the two-year PA intervention, in girls, there was no overweight in the intervention group (vs. 12% overweight in controls) (Figure 5).

Table 3 shows that commencing with the children’s second year at primary school (Sept. 2007, one year after the start of the PA intervention), the odds of being overweight or obese in the intervention children was almost three times lower than that of control children (p < 0.005). Moreover, the odds of the intervention children being overweight or obese in comparison with the controls statistically decreased in a step-wise manner in relation to the duration of the PA intervention: from 0.64 times less after 7 months (April 2007); 0.34 times less at 1 year; 0.16 times less at 1 year 7 months (April 2008); 0.04 times less at 2 years (Sept. 2008). On the other hand, the odds of being overweight or obese in boys was more than two and half higher than in girls before the start of PA intervention (Sept. 2006). However, one year and thereafter after the start of the PA intervention – from Sept. 2007 onwards), the odds of being overweight or obese in boys was not significantly higher in comparison with girls.

## Discussion

We assessed the effectiveness of a school-based two-year PA intervention in reducing obesity and overweight in 6-9-year-old children. As such, the current study bridges the gap between longitudinal studies of school-aged children that focus on the obesity reduction by increased school-based PA in Western countries [33,34], and the lack of such much-needed longitudinal studies in Central/ Eastern European nations.

In terms of the study’s first objective, we described and compared the PA levels of control and intervention girls and boys before, during, and at the end of the PA intervention. Before the PA intervention, there were no differences in PA levels between the intervention and controls on schooldays and on weekends. Across our sample of children (before the intervention) the achieved mean daily steps counts (≈7,700) and AEE (≈9.5 Kcal/Kg·day^-1^) unfortunately did not reach the national PA guidelines for maintaining health for Czech children aged 6–11 years (steps per day – 12,000 girls and 14,000 boys; AEE – 11 Kcal/Kg·day^-1^ for girls and 13 Kcal/Kg·day^-1^ for boys) [36]. The results showed that a higher percentage of intervention girls than intervention boys met the national Czech PA guidelines during the PA intervention programme. Design of PA intervention might score for reduction of the differences in AEE and steps counts between girls and boys. However, the long-term implementation of increased PA within the school environment had a positive impact on the daily PA levels (both step counts and AEE) on schooldays, which among the intervention girls, even reverted to their higher PA levels that they had exhibited at kindergarten [19]. Daily mean step counts of intervention girls and boys exceeded 10,500 during this school-based PA intervention. Despite such increase of ≈ 1133-1485 in terms of daily step counts on schooldays, both our intervention girls and boys lagged behind the levels reported for girls (10,800-14,800) and boys (11,500-18,100) of the same age in Canada, Netherlands, New Zealand, Sweden, the United Kingdom and USA [16,52,53]. As regards the controls, girls’ and boys’ PA levels continuously decreased with repeated monitoring from April 2007 to September 2008. The lowest mean daily steps (< 8,000 girls; < 9,000 boys) and AEE (8 Kcal/Kg·day^-1^ for girls; 9.5 Kcal/Kg·day^-1^ for boys) values were observed at the final monitoring (3^rd^ Grade of primary school). This low level of school PA of controls, in addition to their low weekend PA is not sufficient for maintaining health [36].

As regards objective two, we compared schooldays and weekends PA of control and intervention girls and boys. During weekends, both intervention and control children had significantly lower PA than during schooldays. This is in support of other studies, where lower levels of accelerometer or pedometer-measured weekend PA in comparison to schooldays has been reported in young, school-aged children in England, Mexico and USA [54-56]. Unfortunately, achieving higher school-based PA in our intervention children did not ‘counter’ their decreased PA on weekends (Figures 1,2). This is further supported by the small to moderate correlations (r_P_ = 0.09-0.35) between schooldays and weekends PA levels of our intervention children [assessed by Pearson product–moment correlation coefficient (r_P_) repeatedly before, during, and at the end of intervention]. In our sample, across the duration of the study (2006–2008), we observed a stronger rate of decrease (steeper slope) in daily mean step counts and AEE on weekends than on schooldays, for both genders and both groups of children (Figures 1,2). This finding further highlights the unfavourable (alarming) weekend PA levels of both intervention and control children in relation to the threshold PA levels that are necessary for maintaining health.

As for objective three, we compared school-based and leisure time PA levels of intervention and control girls and boys during schooldays. During the PA intervention, intervention children’s school-based daily mean step counts comprised ≈ 40-44% of their leisure time step counts; whilst the controls’ school-based step counts comprised 25-30% of their leisure time step counts. The intervention children’s school time steps counts (≈3000-3350 per day) corresponded with ≈ 30 minutes of moderate-to-vigorous PA [53] of a value of 4 MET [57]. In contrast, our controls’ mean 1780–1890 steps during school time represented <20 minutes of moderate-to-vigorous PA per day. The increase of ≈ 917-1444 steps during leisure time of schooldays in intervention children represented the equivalent of an increase of ≈ 10-15 minutes of moderate-to-vigorous PA. The basic health-related guidelines for children and youth, independent of their current PA level, is to increase the time spent on moderate-to-vigorous PA by 30 minutes per day; and over a 5 month period, progress to adding an additional 90 minutes of daily PA [58]. In terms of the daily step counts on schooldays, our intervention boys and girls did achieve this guideline.

School time step counts (including steps achieved during after-school nursery) comprised 28-31% of schooldays steps in our intervention children, and 20-23% among our controls. These levels are more modest when compared with findings of previous studies [59,60] where school time step counts represented 44-46% and 43-49% of the daily steps counts in 5-11-years-old girls and boys respectively. Our children’s low level of schooldays PA in comparison with international peers [60], combined with the short distances between their schools and the children’s homes might partially explain the lower percentages of school step counts in relation to results of previous studies [16,60]. However, school breaks’ PA significantly contributed to higher overall schooldays PA of 9- and 10 year-old children [17,18], even for those who were overweight-to-obese [21].

The final monitoring of one-week PA before the end of the PA intervention programme (September 2008) showed a slight decrease of leisure time step counts and AEE (intervention children); and a slight decrease of school time step counts and AEE (controls). These declines of PA could be due to the increased school assignments and homework associated with two subjects that were ‘new’ to the children (English language and basics of humanities and natural science) which are taught to 3^rd^ Grade primary school children in the Czech Republic. These new subjects are associated with increases of regular time-consuming homework (e.g. vocabulary practice with repeated writing of new words and drawing of their sense; drawing of animals, plants and natural objects and phenomena). In addition to the two new subjects, at the start of 3^rd^ Grade of primary school, Czech children need to manage the challenges of grammar of the standard Czech language (e.g. specific rules of spelling of ‘y’, ‘ý’, ‘i’, ’í’, ‘e’, ‘ě’, ‘s’, ‘š’, ‘c’, ‘č’). An impact of such an increase of assignments and homework could have been a decrease of leisure time PA level.

For objective four, we described and compared the proportion of overweight and obese children in the control and intervention girls and boys before, during, and at the end of the intervention; and assessed the effect of participation in the PA intervention on children’s overweight and obesity. Before the start of the PA intervention, there were no differences in the proportions of obese girls and boys of our intervention (7% girls; 11 % boys) and control groups (7% girls; 6% boys). These low levels of obesity at first year of primary school could be due to the well supported PA programmes at kindergartens [22]. The PA intervention was accompanied by significant decreases of overweight and obesity in our intervention girls and boys (Figure 5) i.e. the intervention children were significantly less likely to be overweight and obese when compared with the controls (Table 3). Despite the fact that programmes that combine increased PA and appropriate diet are more effective in obesity reduction in children [10,24,28], our findings indicate that long-term PA in the school environment may also result in a notable reduction of obesity among 7–8 year old children. In line with the conclusions of recent meta-analyses [11,24], we agree that longer-term (>1 year) and content-specific programmes for girls and boys have a higher chance of reducing obesity than shorter-term, non-gender-specific interventions. School support and activity-friendly environments are other prerequisites for the effective implementation of PA interventions.

This study has limitations. The intervention schools were selected on the basis of existing PA-conducive environment, a point that could have contributed to the observed findings, and the non-representativeness of our children to the wider population of children in the Czech Republic requires that caution is exercised when drawing generalisations. In addition, the assessment of body weight level using age-differentiated percentile BMI graphs does not consider issues of body composition or actual ‘biological’ age of the child. We did not monitor the nutritional habits of the children; these could have influenced the rates of overweight and obesity. Other descriptive characteristics of the intervention and control children (socioeconomic status in particular) at baseline would have also been helpful for a more complete assessment of the effectiveness of a school-based two-year PA intervention programme in reducing obesity and overweight in 6–9 year-old children. At present, more comfortable and accurate accelerometers are being used worldwide to monitor children’s PA than the Caltrac accelerometer. Due to the study’s longitudinal design, we used the same kind of accelerometer over the course of the study (2006 – 2008). However, despite these limitations, the longitudinal, repetitive, objectively-monitored PA level simultaneously measured by two devices (pedometer and accelerometer) provides support to the internal validity of the study.

Future research should recruit more schools from more regions/ countries whilst addressing these limitations; and assess the ‘sustainability’/ longevity of the benefits of the intervention on children’s obesity/ overweight levels at a later point in time after the intervention has ended (e.g. after 6 months and 1 year). We monitored PA beginning at school time until the end of the day. Further research would need to assess other potential enhancements of PA levels at other times e.g. before the school day starts (by promoting active school commuting - walking or cycling to school); or alternatively, other activities undertaken during the evenings or weekends (e.g. the role of children’s participation in PA organisations and sports clubs) as means to reduce/prevent obesity and overweight levels. Future studies would also benefit from using electronic devices to access the school environment in relation to children’s PA programme by producing a fine-grained picture (‘minute-by-minute’ records) e.g. ActiGraph accelerometers or heart rate telemetry [17,18], or multi-functional devices [21].

## Conclusions

School-based PA (PE lessons, PA during short breaks and longer recesses, PA at after-school nursery) in compatible active environments (child-friendly gym and school playground, corridors with movement and playing around corners and for games) plays a vital role in overweight and obesity reduction among younger pupils. However, reductions of overweight and obesity levels were observed starting about a year after the PA intervention commenced. Increased school-based PA had also positive impact on leisure time PA of schooldays and on PA at weekends of intervention children. Increased school-based PA during schooldays contributed to: achieving >10,500 steps and >10.5 Kcal/Kg per schoolday across the 2 years of the study; and, led to a stop of the decline in PA that is known to be associated with the increasing age of children. However, despite of the increased school-based PA, the intervention children did not achieve international levels of health maintaining PA.

## Competing interests

The authors declare that they have no competing interests.

## Authors' contributions

ES and DS designed the study. DS, ES and WEA undertook the data analysis. ES and WEA wrote this manuscript with input of DS. All authors approved the final version.

## Pre-publication history

The pre-publication history for this paper can be accessed here:

http://www.biomedcentral.com/1471-2458/12/570/prepub
